# Decreased activity of RCAN1.4 is a potential risk factor for congenital heart disease in a Han Chinese population

**DOI:** 10.1007/s13238-018-0525-8

**Published:** 2018-03-28

**Authors:** Liangping Cheng, Peiqiang Li, He Wang, Xueyan Yang, Huiming Zhou, Wufan Tao, Jie Tian, Hongyan Wang

**Affiliations:** 1 Key Laboratory of Pediatrics in Chongqing, Chongqing International Science and Technology Cooperation Center for Child Development and Disorders, Department of Cardiovascular Medicine, Children’s Hospital of Chongqing Medical University, Chongqing, 400014 China; 2 Obstetrics & Gynecology Hospital, The Institute of Reproduction and Developmental Biology, Fudan University, Shanghai, 200011 China; 3 Institute of Genetics, School of Basic Medical Sciences, Lanzhou University, Lanzhou, 730000 China; 4 The State Key Laboratory of Genetic Engineering, MOE Key Laboratory of Contemporary Anthropology, Collaborative Innovation Center of Genetics and Development, School of Life Science, Fudan University, Shanghai, 200433 China; 5 Institute of Developmental Biology and Molecular Medicine, Fudan University, Shanghai, 200433 China

**Dear Editor,**

Congenital heart disease (CHD) is a major birth defect worldwide. However, the aetiology of CHD remains unclear and the detailed molecular mechanisms underlying CHD pathogenesis have not been fully understood. The human regulator of calcineurin (*RCAN1)* gene is highly expressed in human hearts and brains (Fuentes et al., 1995) and composed of seven exons that are alternatively spliced and/or transcribed by differential promoters to produce different isoforms (Fig. 1A) (Yang et al., 2000). RCAN1.1 (NM_004414) and RCAN1.4 (NM_203418) are the two major isoforms differentially expressed in many tissues and cells. RCAN1.1 is constitutively expressed while the expression of RCAN1.4 is induced by diverse stimuli (Yang et al., 2000; Lange et al., 2004). RCAN1.4 negatively regulates NFAT-mediated transcription (Lange and Yutzey, 2006; Qin et al., 2006), which is an important regulator for early heart development (de la Pompa et al., 1998). Although SNPs rs149048873, rs193289374 and rs143081213 in the RCAN1.1 promoter were not associated with sporadic CHD (Guo et al., 2015; Li et al., 2015), the function of RCAN1.4 in CHD remains elusive.

**Figure 1:**
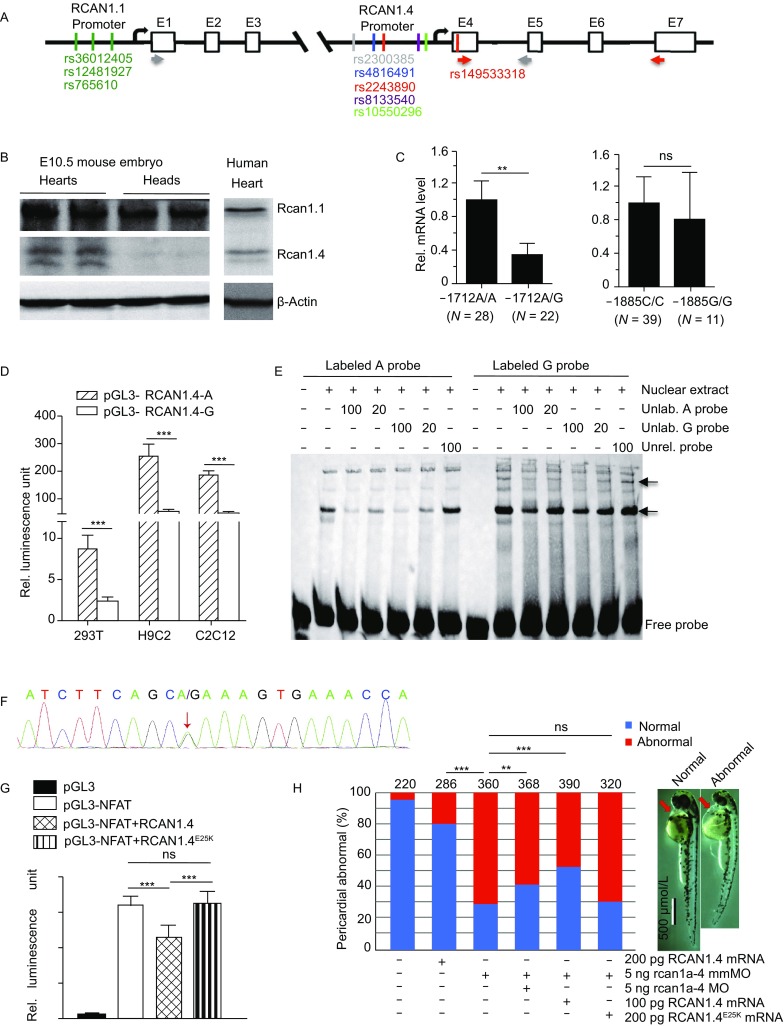
**Decreased activity of RCAN1.4 is a potential risk factor for congenital heart disease**. (A) Schematic structural diagrams of the human *RCAN1* gene and the location of eight SNPs of *RCAN1* identified in this study. Black arrows represent the transcription start sites of RCAN1.1 and RCAN1.4. Grey and red arrows represent the qPCR primers for RCAN1.1 and RCAN1.4, respectively. (B) Western blot of RCAN1.1 and RCAN1.4 proteins in mouse embryonic hearts and heads at embryonic day 10.5 (E10.5) and in a human foetal heart (20 weeks). β-Actin was used as a loading control. The images shown are representative of three experiments with similar results. (C) The minor G allele of rs2243890 attenuates the expression of RCAN1.4 in human heart samples from sporadic CHD patients. qRT-PCR analysis of RCAN1.4 mRNA expression in sporadic CHD patients with -1,712 A/A or A/G alleles of rs2243890 in *RCAN1* gene (left panel) and with −1,885 C/C or G/G alleles of rs2300385 (right panel). The numbers of human heart samples used for qRT-PCR for each genotype were given under each column. GAPDH was used as an internal control. (D) The minor G allele of rs2243890 reduces the activity of the RCAN1.4 promoter in an *in vitro* luciferase reporter assay. The effects of the minor G and major A alleles of rs2243890 on the promoter activity of RCAN1.4 were separately analyzed in the human embryo kidney cells (293T), the rat cardiomyocyte-derived cells (H9C2) and the mouse myoblasts (C2C12) using dual-reporter luciferase system. Values represent the means ± SEM of three separate experiments. Non-parametric tests were used for statistical analysis of the results in (D). (E) The minor G allele probe of rs2243890 displays a higher binding affinity for unknown nuclear proteins from a nuclear extract of 293T cells. The results of an EMSA performed by incubating biotin-labelled minor G and major A allelic probes (G probe and A probe, respectively) of rs2243890 with the nuclear extract from HEK 293T cells in the presence of unlabelled competitor (G probe and A probe, respectively) or non-competitor (unrelated probe) probes. The first arrow indicates a weak, but specific shift band only for labeled G probe. The second arrow indicates a strong shift band with higher affinity to G probe than to A probe. The numbers above the image are the quantity (pm) of unlabeled probes or unrelated probe used for the assay. The Image shown is representative of three experiments with similar results. (F) Sanger sequencing results showing a heterozygous mutation (A/G) at nt 75 (red arrow) in RCAN1.4. (G) Statistical analyses of a luciferase reporter assay in 293T cells transfected with plasmids as indicated, which revealed that this rare mutation abolishes the suppression of RCAN1.4 on NFAT-mediated transcription. (H) The RCAN1.4^E25K^ mutant is a loss-of-function mutation in zebrafish. Right panel: zebrafish embryos with normal or enlarged pericardium after injection of 5 ng rcan1a-4 MO. Red arrows indicate the paricardial cavity of zebrafish embryoes. Left panel: the response frequencies of pericardial defects. Co-injection of 100 pg or 200 pg of wt RCAN1.4 mRNA partially suppressed rcan1a-4 MO-mediated CHD phenotype (enlarged pericardium) while co-injected 200 pg RCAN1.4^E25K^ mRNA has no effect at all. The number above each bar is the total number of embryos examined under each experimental condition. *P* value was calculated by *χ*^2^ analysis. *P < 0.05, **P < 0.01, ***P < 0.001, ns: not significant

To investigate if the RCAN1.4 plays a role in heart development, we evaluated the expression of the RCAN1.1 and RCAN1.4 proteins in E10.5 mouse embryonic hearts and human hearts. The results revealed that both the RCAN1.1 and RCAN1.4 were expressed in mouse embryonic hearts and human hearts (Fig. 1B). In addition, the RCAN1.4 protein was easily detected in embryonic mouse hearts at E10.5 but not in embryonic mouse heads at E10.5, while RCAN1.1 protein levels were similar between embryonic mouse hearts and heads (Figs. 1B and S1A). These results demonstrate that not only RCAN1.4 is expressed in hearts, but also its expression is differentially regulated, at least during mouse heart development, suggesting that the expression level of RCAN1.4 might contribute to human CHD.

To evaluate the association of *RCAN1* gene with human CHD, we performed an association study of variants in *RCAN1* with CHD. The −2 kb of 5′-upstream regulatory regions, coding regions and 3′UTRs for RCAN1.1 and RCAN1.4 transcripts were first sequenced by target-capture sequencing in 412 blood samples of sporadic CHD patients and 213 matched controls from Shandong populations (Table S1). Eight SNPs with a minor allele frequency (MAF) > 0.05 were identified within the −2 kb of 5′ regulatory region of RCAN1.1 and RCAN1.4 transcripts. Three SNPs (rs765610, rs1248192 and rs36012405) and the other five (rs10550296, rs8133540, rs2243890, rs4816491 and rs2300385) were positioned in 5′ regulatory regions of RCAN1.1 and RCAN1.4 transcripts, respectively (Fig. 1A). We also identified one rare mutation (rs149533318, RCAN1.4^E25K^) in the coding region of RCAN1.4. Among them, only the genotype distribution of SNP rs2243890 A>G (HGVS Name: NM_203418:c.-1712 A>G) located in RCAN1.4 promoter was normally different between the CHDs and control (Table S2). We observed the higher frequencies of G allele in CHD (MAF = 0.09) than control (MAF = 0.05) (OR = 1.98, 95% CI = 1.20–3.27, *P* = 0.007 in additive model; OR = 2.11, 95% CI = 1.26–3.53, *P* = 0.004 in dominant model) (Table S2). To further confirm the validity of the association of rs2243890 A>G with CHD, we not only verified the status of rs2243890 A>G by re-sequencing a 0.5-kb genomic sequence around the SNP in the previously analysed 412 sporadic CHD patients and 213 matched controls, but also examined the same sequence in 458 newly collected sporadic CHD patients and 1,107 healthy controls from Shanghai by Sanger sequencing. Consistent results were obtained for this SNP (OR = 1.57, 95% CI = 1.19–2.07, *P* = 0.002) (Table 1). Subsequently, we combined the data from both cohorts for combined analysis and the result showed that the distribution of the minor G allele of rs2243890 was significantly different between the CHD and control groups. The CHD risk was significantly increased by 1.66-fold due to the G allele (OR = 1.66, 95% CI= 1.30–2.11, *P* = 4.68E−05) (Table 1). The frequencies of all genotypes among the control subjects were in accordance with the Hardy-Weinberg expectation (*P* > 0.05). The statistical power of the study is 0.924 at the 0.05 significance level, which is calculated with the power and sample size calculation (version 3.1.2, http://biostat.mc.vanderbilt.edu/wiki/Main/PowerSampleSize).

**Table 1:**
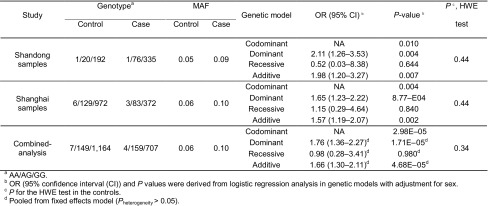
Association of SNP rs2243890 A>G in RCAN1.4 promoter region with CHDs in two independent case-control studies

A further stratified analysis of rs2243890 A>G was performed based on different subtypes of CHDs. The most significant differences in CHD vs. control were observed in an additive genetic model in septal defects, which had a 1.51-fold higher risk in sporadic CHD patients (OR = 1.51, 95% CI = 1.11–2.07, *P* = 0.01) and conotruncal defects, which had a 2.18-fold higher (OR = 2.18, 95% CI = 1.59–2.99, *P* = 1.33E−06) risk in sporadic CHD patients (Table S3). There were no significant associations observed between rs2243890 A>G and other CHD subtypes, including right ventricular outflow tract obstruction (RVOTO) (*P* = 0.12), left ventricular outflow tract obstruction (LVOTO) (*P* = 0.34) and patent ductus arteriosus (PDA) (*P* = 0.41) (Table S3).

Since the minor G allele of rs2243890 (at −1,712 bp) was located in the promoter region for RCAN1.4 transcript, we hypothesized that the G allele may contribute to the risk of CHD by influencing RCAN1.4 transcription. To test our hypothesis, we evaluated the RCAN1.4 mRNA level in human heart tissue samples from sporadic CHD patients by qRT-PCR. We found that RCAN1.4 mRNA level in human heart samples with heterozygous A/G alleles was almost threefold lower than that of samples with homozygous A/A alleles (Fig. 1C), while as a control, the difference in RCAN1.4 mRNA level between the heart samples carrying either heterozygous C/G alleles or homozygous G/G alleles of rs2300385 (at −1,885 bp) was not significant (Fig. 1C). These results suggest that the minor G allele of rs2243890 in *RCAN1* may affect CHD by decreasing rather than enhancing the transcription of RCAN1.4. To further confirm the effect of rs2243890 A>G, we evaluated the RCAN1.4 promoter activity of the A and G alleles of rs2243890 using a dual-luciferase reporter assay in HEK293, H9C2 and C2C12 cells. The results showed that the transcriptional activity of the G allele was significantly lower than that of the A allele in all three cell lines (Fig. 1D), demonstrating that the G allele of rs2243890 attenuates RCAN1.4 transcription. Taken together, these results suggest that the minor G allele of rs2243890 in *RCAN1* may contribute to CHD by inhibiting rather than enhancing RCAN1.4 transcription.

As the minor G allele of rs2243890 decreased RCAN1.4 promoter activity (Fig. 1D), we questioned whether these changes might be due to its different binding affinity for some transcription factors. Using a major probe and a minor probe containing the A allele and G allele, respectively (Table S5), we performed an EMSA to evaluate the binding affinity of these alleles for unknown transcription factors in 293T cells. The results showed that the G allele displayed a higher binding affinity for some nuclear protein(s) than the A allele did (Fig. 1E). As the minor G allele decreased *RCAN1*.4 promoter activity compared with the major A allele, we inferred that the specific binding protein(s) probably function as transcriptional repressors.

By Sanger sequencing we also confirmed the CHD-specific rare mutation RCAN1.4^E25K^ identified from target-capture sequencing (Fig. 1F). The MAF for RCAN1.4^E25K^ in the ExAC database was as rare as 3.35E−05 (4/119506). To assess whether this RCAN1.4^E25K^ mutant plays a role in CHD development, we performed an NFAT promoter-mediated luciferase reporter assay. Results showed that mutant RCAN1.4^E25K^ significantly impaired suppression of *NFAT*-mediated transcription (Figs. 1G and S1B), suggesting that RCAN1.4^E25K^ may be a loss-of-function mutant.

The E25 of human RCAN1.4 protein is conserved from the zebrafish to human (Fig. S1C). About 70% of zebrafishes injected with antisense morpholino-modified oligonucleotides (MO) for zebrafish rcan1a-4 (a zebrafish homolog of human RCAN1.4) (Alghanem et al., 2017) displayed an enlarged pericardium (a CHD phenotype) and this rcan1a-4 MO-mediated CHD phenotype could be partially rescued by co-injected human RCAN1.4 mRNA in a dosage-dependent manner (Fig. 1H). However, human RCAN1.4^E25K^ mRNA failed to rescue rcan1a-4 MO-mediated CHD phenotype, suggesting again that RCAN1.4^E25K^ is a loss-of-function mutation *in vivo*.

In this study, the SNP rs2243890 in RCAN1.4 promoter region was found to be significantly associated with CHD and further validated to be significantly associated with septal and conotruncal defects of CHD by analysing blood samples from 870 sporadic CHD patients and 1,320 normal controls from a Han Chinese population. Our results, therefore, adds one more piece of evidence supporting the conclusion that genetic associations with CHD have a high phenotypic specificity (Soemedi et al., 2012; Cordell et al., 2013) and also suggest a population specificity for genetic association of SNP rs2243890 with CHD because reported GWAS studies did not find the association of SNP rs2243890 with CHD in Caucasian (Cordell et al., 2013).

Consistent with the results from two previous genetic association studies (Guo et al., 2015; Li et al., 2015), we confirmed that three other SNPs (rs765610, rs12481297 and rs36012405) in the RCAN1.1 promoter region were also not associated with CHD. Our Western blot analyses further showed that the RCAN1.4 protein was expressed in both mouse and human embryonic hearts and the RCAN1.4, but not RCAN1.1, protein level was differentially up-regulated in developing mouse embryonic hearts (Fig. 1B). These results support the notion that RCAN1.4, but not RCAN1.1, may contribute to CHD pathogenesis in the Han Chinese population.

*RCAN1,* also known as Down syndrome candidate region 1 (DSCR1), is located in syntenic regions of human chromosome 21. Approximately 40%–60% of DS patients are accompanied by CHD (Stoll et al., 1998; Vis et al., 2009; Elmagrpy et al., 2011). *RCAN1* triplication was thought to be a potential cause for CHD in DS (Lange et al., 2004). However, Lyle et al. identified eight new DS cases of partial monosomy 21. One patient carried only a 1.48 Mb deletion containing eight genes, including *RCAN1*, and displayed a relatively severe DS phenotype, including cardiac anomaly (Lyle et al., 2009). Now, our study revealed that rs2243890 A>G in the RCAN1.4 promoter increases the risk of CHD by decreasing RCAN1.4 transcription. We not only demonstrated that the minor G allele of rs2243890 significantly decreased RCAN1.4 transcription but also showed that the rare mutation RCAN1.4^E25K^ identified in a VSD patient almost completely abolished RCAN1.4 activity in both *in vivo* zebrafish model as well as in an *in vitro* luciferase reporter assay. Altogether, these studies suggest that the expression or activity of RCAN1, rather than the *RCAN1* copy number, may contribute to DS-associated CHD. Our study may provide a new understanding of the role of the *RCAN1* gene in human CHD.

## Electronic supplementary material

Below is the link to the electronic supplementary material.

Supplementary material 1 (PDF 751 kb)
