# Supplementary material for: Decreased activity of RCAN1.4 is a potential risk factor for congenital heart disease in a Han Chinese population
Source: Protein Cell. 2018 Mar 28;9(12):1039–44. doi: 10.1007/s13238-018-0525-8 (PMC6251805; doi:10.1007/s13238-018-0525-8)
Supplement: Supplementary file 1 — Supplementary material 1 (PDF 751 kb) [file 13238_2018_525_MOESM1_ESM.pdf]

Supplemental Figure 1

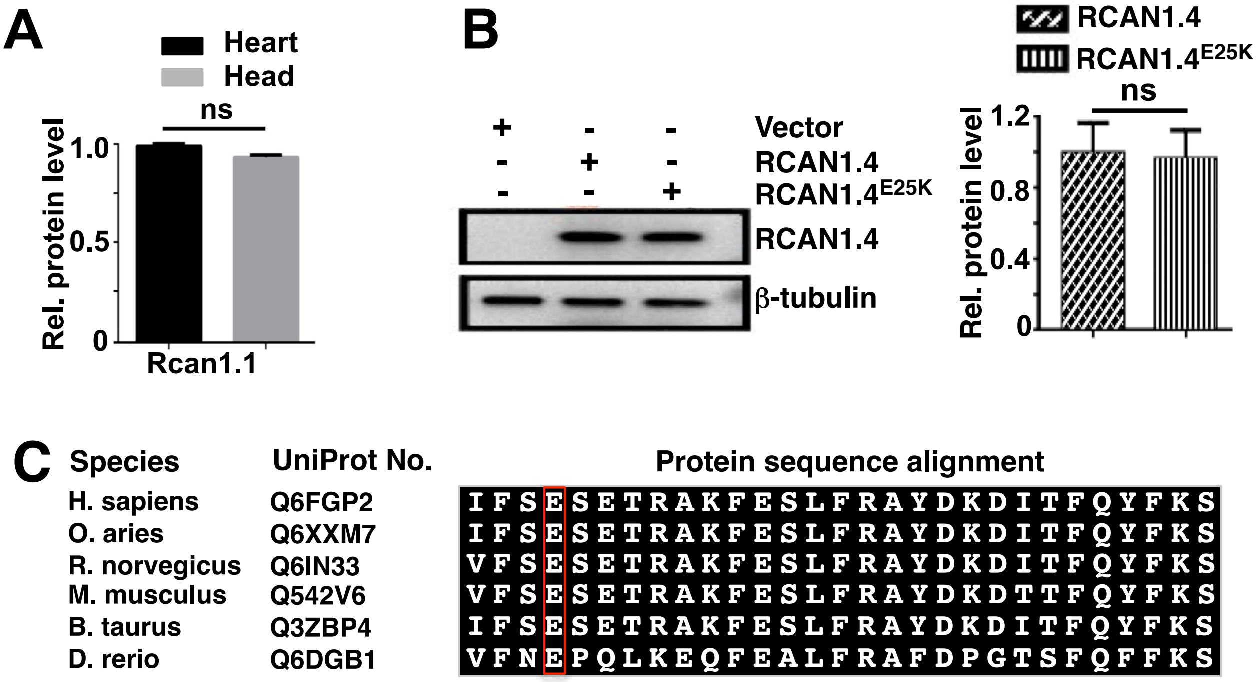

A: Statistical analyses of the RCAN1.1 protein in mouse embryonic hearts and heads at embryonic day 10.5 detected in Western blot in Fig. 1B of main text. **B:** Western blot (left panel) and statistical analyses (right panel) of exogenous RCAN1.4 protein detected with an anti-HA tag in the cell lysates used for assay for Fig. 1G in main text. β-tubulin was used as a loading control. The images shown are representative of three experiments with similar results. Values in (A and B) represent the means ± SEM of three separate experiments. Non-parametric tests was used for statistical analysis. ns, not significant. **C:** Alignment of partial amino acid sequences of RCAN1.4 orthologous proteins. The amino acid that was mutated in a VSD patient is boxed; UniProt No.: protein accession number in the UniProt database.

Supplemental Tables

Table S1 Demographic characteristics in CHD cases and controls

Variable	Cases (%)	Controls (%)	*p* value
Shandong Group			
Age, years (mean±SD)	2.95±2.7	7.1±3.7	p<0.05
Gender			
Male	229(55.6%)	106(49.8%)	0.19
Female	183(44.4%)	107(50.2%)	
Shanghai			
Age, years (mean±SD)	2.84±4.09	30.05±10.78	p<0.05
Gender			
Male	233(50.8%)	625(56.4%)	0.05
Female	225(49.2%)	482(43.6%)	
CHD classification			
Septation defects	326(37.5%)		
Conotruncal defects	247(28.4%)		
RVOTO	86(9.9%)		
PDA defects	53(6.1%)		
LVOTO defects	53(6.1%)		
AVSD	33(3.8%)		
APVR defects	25(2.9%)		
Heterotaxy defects	14(1.6%)		
Complex defects	14(1.6%)		
Others	19(2.2%)		
Total	870(100%)		

RVOTO, Right Ventricular Outflow Tract Obstruction;

PDA, patent ductus arteriosus;

LVOTO, Left Ventricular Outflow Tract Obstruction;

AVSD, Atrioventricular Septal Defect;

APVR, Anomalous pulmonary venous return.

Table S2 Summary of the common variants of *RCAN1* identified by target-captured sequencing

RCAN1	Transcript ID	Location	Genotype		MAF			Genetic model	OR (95% CI) [c]	*P* value [c]	*P* [d], HWE test
			Control[b]	Case[b]	Control	Case	Database[e]				
**rs10550296 -/TCAA** [a]	NM_203418	Intron	2/27/183	2/82/328	0.07	0.10	0.10	Genotypic	NA	0.060	0.30
								Dominant	1.64(1.03-2.60)	0.035	
								Recessive	0.52(0.07-3.70)	0.510	
								Additive	1.51(0.98-2.34)	0.062	
**rs8133540 C/A** [a]	NM_203418	Intron	13/77/123	29/149/233	0.24	0.25	0.29	Genotypic	NA	0.120	0.85
								Dominant	1.03(0.74-1.44)	0.865	
								Recessive	1.12(0.57-2.22)	0.736	
								Additive	1.04(0.79-1.36)	0.786	
**rs2243890 G/A** [a]	NM_203418	Intron	1/20/192	1/76/335	0.05	0.09	0.03	Genotypic	NA	0.010	0.44
								Dominant	2.11(1.26-3.53)	0.004	
								Recessive	0.52(0.03-8.38)	0.644	
								Additive	1.98(1.20-3.27)	0.007	
**rs4816491 T/C** [a]	NM_203418	Intron	2/32/179	2/60/348	0.08	0.08	0.09	Genotypic	NA	0.760	0.65
								Dominant	0.92(0.58-1.45)	0.719	
								Recessive	0.49(0.07-3.51)	0.477	
								Additive	0.90(0.59-1.38)	0.625	
**rs2300385 C/G** [a]	NM_203418	Intron	23/90/100	53/192/166	0.32	0.36	0.41	Genotypic	NA	0.300	0.75
								Dominant	1.30(0.93-1.82)	0.124	
								Recessive	1.19(0.71-2.01)	0.520	
								Additive	1.20(0.94-1.54)	0.149	

**rs765610 C/T** [a]	NM_00 4414	5' upstream	39/112/61	85/201/124	0.45	0.45	0.46	Genotypic	NA	0.690	0.40
								Dominant	0.93(0.65-1.35)	0.716	
								Recessive	1.15(0.75-1.75)	0.530	
								Additive	1.02(0.80-1.29)	0.904	
**rs12481927 T/C** [a]	NM_00 4414	5' upstream	18/98/97	41/195/176	0.31	0.34	0.40	Genotypic	NA	0.720	0.43
								Dominant	1.13(0.81-1.57)	0.490	
								Recessive	1.20(0.67-2.14)	0.547	
								Additive	1.11(0.86-1.44)	0.420	
**rs36012405 G/C** [a]	NM_00 4414	5' upstream	50/117/45	94/197/118	0.51	0.47	0.47	Genotypic	NA	0.120	0.17
								Dominant	0.67(0.45-0.99)	0.047	
								Recessive	0.97(0.66-1.44)	0.895	
								Additive	0.85(0.67-1.08)	0.185	

a Major allele

b Homo Minor/Heterogous/Homo Major

c Adjusted by sex

d HWE test in the controls

e MAF, minor allele frequency from 1000 Genomes (EAS: East Asian)

Table S3 Stratification analysis of rs2243890 A>G in *RCAN1.4* Promoter region according to CHD classification and phenotype

CHD Classification	Genotyping in Cases [a]	Genetic model	OR (95% CI) [b]	*P*-value[b]
Septation defects	267/59/0	Codominant	NA	NA
		Dominant	**1.65(1.19-2.28)**	**0.003**
		Recessive	NA	NA
		Additive	**1.51(1.11-2.07)**	**0.010**
Conotruncal defects	188/57/2	Codominant	NA	**3.08E-06**
		Dominant	**2.38(1.70-3.34)**	**4.97E-07**
		Recessive	**1.64(0.34-7.95)**	0.543
		Additive	**2.18(1.59-2.99)**	**1.33E-06**
RVOTO	70/16/0	Codominant	NA	NA
		Dominant	1.70(0.96-3.01)	0.07
		Recessive	NA	NA
		Additive	1.53(0.90-2.61)	0.12
LVOTO	49/4/0	Codominant	NA	NA
		Dominant	0.62(0.22-1.74)	0.37
		Recessive	NA	NA
		Additive	0.61(0.22-1.68)	0.34
PDA	45/7/1	Codominant	NA	0.54
		Dominant	1.28(0.59-2.78)	0.53
		Recessive	3.10(0.364-26.3)	0.30
		Additive	1.34(0.67-2.66)	0.41

a AA/AG/GG;

b OR_{add} (95% confidence interval (CI)) and *P* values were derived from logistic regression analysis in genetic models with adjustment for sex; NA, not available; Note: The total 1320 combined controls were used for analysis

Table S4

Sample No.	Sampling location	Diagnosis phenotype	Gender	Age (year)	Genotype	
					rs2243890	rs2300385
1	right atrium	VSD	female	1	T/T	C/C
2	auricula dextra	CoA+VSD+PH	male	0.1	T/T	C/C
3	right atrium	VSD	female	7	T/T	C/C
4	right atrium	VSD+PDA+PH	male	1.9	T/T	C/C
5	right atrium	TOF	male	2.2	T/T	C/C
6	right atrium	ASD+PS	male	6	T/C	C/C
7	right atrium	ASD	male	0.75	T/T	C/C
8	right atrium	TOF	female	10	T/C	G/G
9	right atrium	TOF	female	1	T/C	G/G
10	right atrium	ASD+PH	female	0.25	T/C	C/C
11	right atrium	PDA	male	0.25	T/C	G/G
12	right atrium	DORV+PS	male	0.5	T/C	G/G
13	right atrium	TOF+PA	male	1.5	T/C	C/C
14	right atrium	TOF	male	1.3	T/C	C/C
15	right atrium	TAPVC	male	0.2	T/C	G/G
16	right atrium	VSD、PH	male	0.8	T/T	C/C
17	right atrium	VSD、ASD	male	0.2	T/C	G/G
18	right atrium	TAPVC	male	0.42	T/C	G/G
19	right atrium	TOF	female	5	T/C	C/C
20	right atrium	PDA、MI	female	2.1	T/C	C/C
21	auricula dextra	DORV、PH	male	0.3	T/C	C/C
22	right atrium	VSD、PH	female	5	T/C	C/C
23	right atrium	MI、TI、PH	female	7	T/C	C/C
24	right atrium	VSD	female	1.75	T/C	C/C
25	right atrium	DORV、SV、PS	male	4	T/T	C/C
26	right atrium	ASD	female	3.42	T/T	G/G
27	right atrium	VSD	male	6	T/C	C/C
28	right atrium	VSD、PH	male	1	T/C	C/C
29	right atrium	VSD、ASD、PH	male	0.25	T/T	C/C
30	auricula dextra	VSD	male	1.67	T/T	C/C
31	auricula dextra	VSD、TI、PDA、PH	female	1.58	T/T	C/C
32	auricula dextra	VSD、PFO、PH	male	0.1	T/T	C/C
33	right atrium	AI	male	0.8	T/T	C/C
34	auricula dextra	VSD、MI、PH	male	0.1	T/C	C/C
35	right atrium	TOF	female	2	T/C	C/C
36	right atrium	VSD	male	1.95	T/T	C/C
37	right atrium	c-TGA、PS	female	0.75	T/T	C/C
38	right atrium	VSD	female	7	T/T	C/C
39	right atrium	VSD	female	3	T/T	C/C
40	right atrium	VSD	male	6	T/T	C/C
41	right atrium	TOF	female	9	T/T	C/C
42	auricula dextra	VSD、PH	male	0.5	T/T	C/C
43	right atrium	PS	male	2	T/T	C/C
44	right atrium	TOF	male	1.8	T/T	C/C
45	right atrium	DORV+PA	female	2.25	T/T	C/C
46	auricula dextra	VSD、ASD、PH	male	0.25	T/T	C/C
47	auricula dextra	VSD	male	0.67	T/C	G/G
48	right atrium	VSD、PFO	male	0.83	T/T	C/C
49	right atrium	TOF	male	0.75	T/T	C/C
50	auricula dextra	TOF	male	0.25	T/T	C/C

Note:ASD: atrial septal defect, CoA: Coarctation of the aorta, DORV: double outlet right ventricle, TAPVC: Total anomalous pulmonary venous connection, PDA: patent ductus arteriosus, PFO: patent foramen ovale, PH: pulmonary arterial hypertension, PS : pulmonary stenosis, SV: single ventricle,TI: tricuspid regurgitation,TOF:tetralogy of fallot, VSD:Ventriculap Septal Defect AI:aortic valve insufficiency, c-TGA： complete transposition of the great arteries, MI: mitral valve insufficiency

Table S5 Primers and probes used in the study

Oligo name	Sequence
RCAN1.4 CDS cloning F	AACCATAACATTCCCCGACCT
RCAN1.4 CDS cloning R	GGGTAATCCGCTTCGCTTG
Point mutation (rs2243890) F	TTGATGCATGTTGCACTGTTCAGTATTCTAC
Point mutation (rs2243890) R	GTAGAATACTGAACAGTGCAACATGCATCAA
Point mutation (rs149533318) F	TGATATCTTCAGCAAAAGTGAAACCAG
Point mutation (rs149533318) R	CTGGTTTCACTTTTGCTGAAGATATCA
RCAN1.4 promoter F	GGGGTACCCCGCCTCTTCTGTCTCTTCTGA
RCAN1.4 promoter R	CCGCTCGAGCGGCTTGCTTTCTTACAGTGAAA
RCAN1.4 Genotyping F	GCCTCTTCTGTCTCTTCTG
RCAN1.4 Genotyping R	GGATGAGTGAGTGTTGCTTA
RCAN1.4 q-PCR F	GGGTCTGTAGCGCTTTCAC
RCAN1.4 q-PCR R	GTTCAGCTGAGGTGGATCGGCGTG
RCAN1.1 q-PCR F	ATGGAGGACGGCGTGGCCGGTCCC
RCAN1.1 q-PCR R	TCCTATGTGTAAGGTCTGAGCA
GAPDH F	TCTGGATCCTCACCACCATGGAGAAGGC
GAPDH R	ATACTCGAGGCAGGGATGATGTTCTG
labeled A -1712 for EMSA F	Biotin- TTCCCCTTGGTGTTTGATGCATGTTGCAT TGTTCAGTATTCTAC
labeled A -1712 for EMSA R	Biotin- GTAGAATACTGAACAATGCAACATGCAT CAAACACCAAGGGGAA
labeled G -1712 for EMSA F	Biotin- TTCCCCTTGGTGTTTGATGCATGTTGCAC TGTTCAGTATTCTAC
labeled G -1712 for EMSA R	Biotin- GTAGAATACTGAACAGTGCAACATGCAT CAAACACCAAGGGGAA
non-specific for EMSA F	AGAGTGTTGTCTGAGTAGCTGAATGGAA
non-specific for EMSA R	TTCCATTCAGCTACTCAGACAACACGCT

Supplemental Materials

Materials and Methods

Study subjects

Blood samples from 820 sporadic CHD patients were collected, among which 412 samples were collected from the Cardiovascular Disease Institute of Jinan Military Command (Shandong, China) and 408 samples were collected from the Children's Hospital of Fudan University, Shanghai. Sporadic CHD cases were diagnosed by echocardiography with some diagnoses further verified by surgery. Patients with additional clinical features including developmental anomalies and positive family history of CHD in a first-degree relative were excluded. A total of 1320 control human blood samples were collected from ethnic- and gender-matched, unrelated healthy volunteers recruited from two corresponding geographical areas (213 from Shandong and 1107 from Shanghai). All subjects were genetically ethnic Han Chinese. All of the CHD cases were classified according to a method previously described (Botto et al., 2007) (detailed diagnostic information on the patients is shown in Suppl. Table 1). Fifty heart tissue samples from sporadic CHD patients were obtained from the children who underwent cardiac surgery in the Children's Hospital of Fudan University, Shanghai for Quantitative real-time PCR (qRT-PCR) analysis. The studies on our samples were conducted in accordance with the Declaration of Helsinki. The protocols used in this work were reviewed and approved by the Ethics Committee of the School of Life Sciences, Fudan University. Written consent was obtained from the parents or guardians of the children.

DNA sequencing and genotyping

The genomic structures of human *RCAN1* genes were determined using human genome assembly GRCh37/hg19. The -2000 bp of 5'-upstream regulatory regions, coding regions and 3'UTR for RCAN1.1 and RCAN1.4 transcripts were sequenced by next-generation sequencing. Genomic DNA-fragment libraries and target enrichment using a probe specific for *RCAN1* were carried out as previously described

(Li et al., 2017). The variants were called using SAMTools(Li et al., 2009) and the variants with a quality score below 1.5 were excluded based on the hg19/GRCh37 database. Known variants were assigned respective SNP codes as dbSNP137. The SNP with high missing genotype rates (<90%) and deviating from Hardy-Weinberg equilibrium in the controls (p<0.05) were also eliminated. Finally, eight common SNPs in *RCAN1* were identified and further analysed. To confirm the genotyping results from next generation sequencing, ~25% of the samples were randomly selected for Sanger sequencing on rs2243890 and rs2300385 and results were 92% concordant. The primers for PCR/DNA sequencing are provided in Suppl. Table 5. For the validation stage, we sequenced the PCR-amplified 0.5kb DNA fragment containing rs2243890 for all samples (870 CHD and 1320 Controls). About 10% of the samples (220) were randomly selected to be genotyped again by Sanger sequencing and the results were 100% concordant.

Quantitative RT-PCR

Total RNA was extracted from human heart tissues of CHD patients and qRT-PCR analyses for RCAN1.1 and RCAN1.4 mRNA were performed as previously described (Yu et al., 2016). The primers for qRT-PCR are listed in Suppl. Table 4.

Plasmid, cell culture, Western blot and luciferase reporter assays

The luciferase reporter plasmid pGL3-RCAN1.4-A was constructed by cloning a 2041bp PCR fragment (-1 to -2041, the first nucleotide before initial ATG of RCAN1.4 as -1) containing the major A allele of rs2243890 into pGL3-Basic reporter vector (Promega E1751). pGL3-RCAN1.4-G was derived from pGL3-RCAN1.4-A using *in vitro* site-specific mutagenesis. The pCMV6-RCAN1.4 was constructed by cloning an RCAN1.4 cDNA into pCMV6-Ac-HA. pCMV6-RCAN1.4^{E25K} was derived from pCMV6-RCAN1.4 using *in vitro* site-specific mutagenesis.

Transfection was performed in 24-well plates with Lipofectamine 2000. To analyse the effect of rs2243890 of the RCAN1.4 promoter, 293T, C2C12 and H9C2 cells were transfected with 200ng pGL3-RCAN1.4-A or pGL3-RCAN1.4-G per well. To assess

the function of RCAN1.4^{E25K} mutation, HEK 293T cells were transfected with 200ng pGL3-NFAT (Addgene 17870) and 50ng RCAN1.4 or pCMV6-RCAN1.4^{E25K} per well. pRL-TK plasmid (5 ng/well) was added as internal control. The dual-luciferase reporter assay was performed 24 hr post-transfection according to the manufacturer's instructions.

Sixteen mouse embryonic heads and dissected hearts at E10.5 and a human foetal heart from an abortion were homogenized and lysed in lysis buffer (50mM Tris HCl, pH 7.4, with 150mM NaCl, 1mM DTA, and 1% Triton X-100) followed by Western blot analysis as describe previously(Li et al., 2017) using antibodies against RCAN1 (Sigma, D6694), β-actin (Sigma, A1978), and β-tubulin.

Probe design and EMSA

We used two online bioinformatic algorithms to predict the effect of genetic variants in the RCAN1.4 promoter: Alibaba (http://www.gene-regulation.com/pub /programs/alibaba2/index.com) and TFsearch (www.cbrc.jp/research/db /TFSEARCH.html). Biotin-labelled minor G and major A allele probes (32 bp corresponding to -1696 to -1727 of RCAN1.4, see Suppl. Table 5) were commercially synthesized (Huajin, Shanghai) and used for EMSA. Nuclear extracts were prepared from HEK 293T cells using the NE-PER Nuclear and Cytoplasmic Extraction kit (Thermo Fisher Scientific, USA). EMSA assay was performed according to the procedures provided in the EMSA kit (Thermo Fisher Scientific).

Zebrafish injection

Studies were undertaken using AB strain zebrafish (*Danio rerio*) under standard conditions at 28℃. 5ng rcan1a-4 MO (morpholino antisense to zebrafish rcan1a-4 start-codon region) or 5ng rcan1a-4 mmMO (mismatched morpholino antisense to zebrafish rcan1a-4 start-codon region) at volume of 2.3 nl were injected into each zebrafish embryo at one- to two-cell stage. Zebrafish rcan1a-4 morpholinos were purchased from Gene Tools and the sequences are rcan1a-4: ACTTCATTGTTTTCAGGTGCATGAC and mismatch control:

ACaTgATTcTTTTgAGcTGCATGAC (Alghanem et al., 2017). For rescue (add-back) study, plasmids (pCMV6-AC-HA-RCAN1.4 WT and pCMV6-AC-HA-RCAN1.4^{E25K}) were linearized by Pme I (NEB) and the mRNAs were *in vitro* transcribed with mMESSAGE mMACHINE™ T7 (Invitrogen: 00439077). The mRNAs were diluted in RNA-free water. Then, Then, 100pg or 200pg human RCAN1.4 or 200pg human RCAN1.4^{E25K} were co-injected with 5ng rcan1a-4 MO into each zebrafish embryo at one to two-cell stage. Forty eight hrs. post-injection, photographs were taken of the harvested embryos using a Zeiss 79005 Zen microscope and their morphology was grossly analysed.

Statistical analysis

Hardy-Weinberg equilibrium (HWE) was examined using the chi-squared test for the controls. Odds ratios (ORs) and 95% confidence intervals (CI) and *p* values were calculated using unconditional logistic regression analysis in an additive genetic model with adjustment for sex. The analyses of the association between different genotypes and CHD risk were performed using the Plink1.9 software. The combined data from Shandong and Shanghai cohorts were also studied by meta-analysis under a fixed-effects model to estimate their combined effect. Confidence intervals (CI) for odds ratios (OR) of meta-analysis were calculated with a Meta package in R (http://cran.r-project.org/web/packages/meta/index.html). Except for those as indicated, all statistical tests were two-tailed, with $p<0.05$ set as the significance level. *$p<0.05$, **$p<0.01$, ***$p<0.001$.

References:

Alghanem, A.F., Wilkinson, E.L., Emmett, M.S., Aljasir, M.A., Holmes, K., Rothermel, B.A., Simms, V.A., Heath, V.L., and Cross, M.J. (2017). RCAN1.4 regulates VEGFR-2 internalisation, cell polarity and migration in human microvascular endothelial cells. Angiogenesis 20, 341-358.

Botto, L.D., Lin, A.E., Riehle-Colarusso, T., Malik, S., Correa, A., and National Birth Defects Prevention, S. (2007). Seeking causes: Classifying and evaluating congenital heart defects in etiologic studies. Birth Defects Res A Clin Mol Teratol 79, 714-727.

Li, H., Handsaker, B., Wysoker, A., Fennell, T., Ruan, J., Homer, N., Marth, G.,

Abecasis, G., Durbin, R., and Genome Project Data Processing, S. (2009). The Sequence Alignment/Map format and SAMtools. Bioinformatics 25, 2078-2079.
Li, P., Huang, L., Zheng, Y., Pan, X., Peng, R., Jiang, Y., Finnell, R.H., Li, H., Qiao, B., and Wang, H.Y. (2017). A missense mutation in TCN2 is associated with decreased risk for congenital heart defects and may increase cellular uptake of vitamin B12 via Megalin. Oncotarget 8, 55216-55229.
Yu, L.W., Wang, F., Yang, X.Y., Sun, S.N., Zheng, Y.F., Li, B.B., Gui, Y.H., and Wang, H.Y. (2016). Mild decrease in TBX20 promoter activity is a potentially protective factor against congenital heart defects in the Han Chinese population. Sci Rep 6, 23662.
